# Peripheral giant cell granuloma associated with a dental implant: A case report

**DOI:** 10.4317/jced.57189

**Published:** 2021-10-01

**Authors:** Alba Sánchez-Torres, Berta Pérez-Amate, Alberdi-Navarro Javier, Iñaki Cercadillo-Ibarguren, Rui Figueiredo, Eduard Valmaseda-Castellón

**Affiliations:** 1 DDS, MS, Master of Oral Surgery and Implantology. Associate Professor of Oral Surgery, School of Medicine and Health Sciences, University of Barcelona. Researcher at the IDIBELL Institute. Barcelona, Spain; 2 DDS, Fellow of Master of Oral Surgery and Implantology. School of Medicine and Health Sciences, University of Barcelona, Spain; 3 DDS, MS, PhD, Oral Medicine and Oral and Maxillofacial Pathology Units, Dental Clinic Service. Department of Stomatology II. University of the Basque Country (UPV/EHU). Leioa, Spain; 4 DDS, MS, PhD, Master of Oral Surgery and Implantology. Associate Professor of Oral Surgery, School of Medicine and Health Sciences, University of Barcelona, Barcelona. Researcher at the IDIBELL Institute. Barcelona, Spain; 5 DDS, MS, PhD, Master of Oral Surgery and Implantology. Professor of Oral Surgery, School of Medicine and Health Sciences, University of Barcelona, Barcelona. Researcher at the IDIBELL Institute. Barcelona, Spain; 6 DDS, MS, PhD, EBOS. Professor of Oral Surgery, Professor of the Master of Oral Surgery and Implantology. School of Medicine and Health Sciences, University of Barcelona. Researcher at the IDIBELL Institute. Barcelona, Spain

## Abstract

Peripheral giant cell granuloma (PGCG) is a reactive exophytic lesion classified as a benign tumor of the oral mucosa. Although its etiology is not clear, it may be a consequence of local chronic irritation or persistent trauma. The objective of this case report was to document the main clinical and histopathological characteristics of a patient with a PGCG associated with a dental implant. A 36 years-old man presented a partly-ulcerated violet-colored sessile-based tumor in the buccal aspect of an implant placed in the fourth quadrant. Radiographically, the implant had one third of marginal bone loss. Differential diagnosis included PGCG and pyogenic granuloma. The implant and the lesion were removed and the histopathological diagnosis was PGCG. After 6 months, there was no evidence of relapse. Peripheral giant cell granulomas may appear in implants that have suffered bone loss. When facing with peri-implant soft tissue lesions, it is advisable to perform an anatomopathological study to obtain a correct diagnosis, to establish an adequate treatment plan, and to rule out malignant lesions.

** Key words:**Peri-implant bone loss, peripheral giant cell granuloma, benign tumor.

## Introduction

Peripheral giant cell granuloma (PGCG) is a reactive exophytic lesion classified as a benign tumor of the oral mucosa (1) that originates from the periosteal cells or the periodontal ligament (2). Its etiology is unclear, although it seems to be related to a local persistent trauma (3-5). The lesion can also be called giant cell epulis, osteoclastoma, reparative giant cell granuloma, or giant cell hyperplasia (1). The differential diagnosis should include pyogenic granuloma since the symptoms and macroscopic features are similar: a fleshy, soft, sessile or pedicle tumor with a color that ranges from dark red to purple, located in the attached gingiva or in the mucosa of the alveolar ridge (2,4).

PGCG appears more frequently in the posterior mandibular region (6). It can be diagnosed at any age, although the maximum incidence is observed between the fifth and sixth decades of life with a slight predominance for the female gender (3,6). Clinically, this lesion tends to bleed and compromise the underlying alveolar bone (3,7,8). Clinically, a mass with variable consistency (soft-firm), with a shiny nodular appearance or a sessile, bluish mass with a shiny surface is observed. Dimensions can range from a small papule to an elongated mass, located in the interdental papilla (2,4).

Despite being a benign lesion, the relapse rate is high even when a total excision of the lesion with curettage is performed (9). Although it is infrequent (10,11), PGCG can appear associated with dental implants and some papers have recommended implant removal to prevent further recurrences (6). The fact that less than 40 cases (6) of peri-implant PGCGs have been published in the literature, might raise some doubts regarding the possible differences between the etiopathogenic characteristics of lesions associated or not to dental implants.

The objective of this paper was to describe the main clinical and histopathological features of a patient with a peripheral giant cell granuloma associated with a dental implant.

## Case Report

A 36-year-old male patient with an asymptomatic lesion associated with an implant placed in right lower first molar was seen in a private dental practice. The patient had a history of ulcerative colitis (under treatment with mesalazine 1g), but reported no allergies, surgical procedures or toxic habits. He also had a gingivitis induced by dental plaque and he brushed his teeth 2 times a day (0-1).

A lesion located in the buccal aspect of the implant (unknown brand) that appeared approximately one month before the appointment could be observed in the clinical examination. The patient referred occasional bleeding when brushing but he did not describe any other symptoms. The implant had been placed 2 years before and was restored with a cemented metal-ceramic crown. No follow-up or implant maintenance appointments have been made since then.

**Figure 1:**
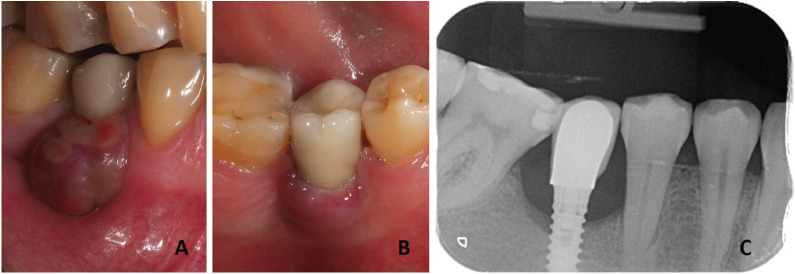
Clinical and radiological characteristics. A) Partially-ulcerated violet-colored sessile-based tumor on the buccal peri-implant mucosa, B) Lingual aspect of the lesion, C) Periapical radiography were marginal bone loss can be observed.

The lesion was a partly-ulcerated violet-colored sessile-based tumor that mainly affected the buccal peri-implant mucosa. Radiographically, the implant had a marginal bone loss of approximately one third of its length (Fig. 1). At that moment, the differential diagnosis included PGCG and pyogenic granuloma.

**Figure 2:**
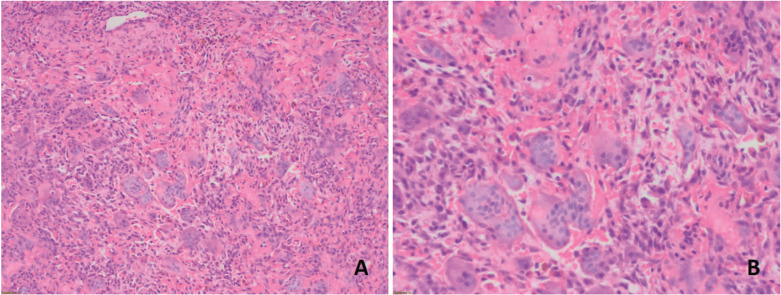
Histopathological exam of the incisional biopsy. Fibrocellular connective tissue with a proliferation of mononuclear ovoid or spindle-shaper cells intermingled by osteoclast-type multinucleated cells in a vascular background A) Hematoxylin and eosin stain (H&E) with a 20x magnification, B) H&E with a 40x magnification.

A first incisional biopsy of the buccal area of the tumor was performed. The specimen was immersed in a 10% formaldehyde solution and was sent to the Oral and Maxillofacial Pathology and Diagnosis Service (SDPOMF, Bizkaia, Spain) for analysis. The histopathological study of the lesion revealed a non-encapsulated proliferation of mononuclear ovoid or spindle-shaper cells intermingled by osteoclast-type multinucleated cells in a vascular background (Fig. 2). A diagnosis of PGCG was established. At that moment, blood levels of parathormone, calcium, phosphorous and alkaline phosphatase were requested in order to rule out any hormonal alteration. Since all these parameters were within physiological ranges, a surgical removal of the lesion that included the periosteum, a healthy mucosal margin, and the implant removal was performed under local anesthesia with 4% articaine and 1:100.000 adrenaline (Artinibsa; Inibsa Dental, Lliça de Vall, Spain). The wound was covered with a collagen dressing and a 4/0 monofilament suture was placed. After 7 days, the area was healing correctly and the sutures were removed. Once again, the histopathological exam confirmed the PGCG diagnosis. Six months after surgery, the patient showed a complete soft tissue healing without any evidence of relapse.

## Discussion

PGCG associated with dental implants are an uncommon finding (3). As a consequence of the few cases documented to date, the etiology and incidence of these lesions has not been fully defined (3,4,8,11). The etiology of this entity seems to be associated with a chronic local irritation of the peri-implant soft tissues (2,4). It is likely that, plaque accumulation or the presence of foreign bodies such as cement remains could be related with the majority of PGCG associated with dental implants (3,8,12).

In patients without implants, PGCG seems to be more common in women (6,9). Some authors attribute it to the action of certain hormones (estrogens or progesterone) that could be involved in the appearance and development of the lesion (3).

The most frequent location, as observed in the present case, seems to be the posterior region of the mandible (1-4). Chrcanovic *et al*. (6) relate this finding to the difficulty in maintaining a correct oral hygiene in this area.

Ogbureke *et al*. (1) claimed that this type of lesions do not involve the underlying bone, since they are a soft tissue pathology. Others, such as Özden *et al*. (5), considered that bone loss could enhance biofilm adhesion to the rough surface of the implant, which in turn would exert a chronic irritating effect on the peri-implant mucosa, leading to the formation of a PGCG.

The treatment performed in the present case was based on the complete removal of the lesion with safety margins and an adequate curettage of the underlying alveolar bone to avoid possible relapses. Any irritating factors that may be causing or favoring the appearance of PGCG must also be eliminated, and it may even be necessary to remove any implant that exerts a traumatic effect or impedes the complete removal of the lesion (4,7,12,13).

Houston (14) and Bukers and White (15), determined that the initial presentation of PGCG may be secondary to some type of hormonal alteration, so clinicians should request blood tests to determine the levels of calcium and alkaline phosphatase to rule out hyperparathyroidism or a parathyroid adenoma.

Despite being a benign lesion, the relapse rates are higher when it appears associated with dental implants. According to a study published by Chrcanovic *et al*. (6) this issue might be related to the persistence of irritating factors such as the presence of biofilm or residual cement. Thus, the removal of the lesion should be complemented with additional therapy such as curettage or peripheral ostectomy to ensure a correct cleaning of the area (6). In this same direction, Peñarrocha-Diago *et al*. (3) did not obtain relapses after 1 year of follow-up when a surgical treatment with a ressective approach and implantoplasty was performed. Overall, many authors agree that peri-implant PGCG are underestimated possibly because some professionals remove inflamed peri-implant tissues without performing histopathological analysis (4,8).

An optimal implant placement and a correct prosthetic design that allows an adequate access to oral hygiene is essential to prevent the occurrence of inflammatory or reactive injuries (8).

## Conclusions

Peripheral giant cell granulomas may be associated with dental implants that have suffered bone loss. When peri-implant soft tissue lesions are observed, an anatomopathological study should be made to obtain a correct diagnosis and to establish an adequate treatment plan, therefore reducing the relapse rate and assuring that malignant lesions are ruled out.
